# Environmental Bridgebuilding: Connecting People Through Nature Conservation. The Example of the Barn Owl

**DOI:** 10.1002/ece3.73795

**Published:** 2026-07-06

**Authors:** Christine Mohr, Philippe Le Billon, Yossi Leshem, Vasileios Bontzorlos, Alexandre Roulin

**Affiliations:** ^1^ Institute of Psychology University of Lausanne Lausanne Switzerland; ^2^ Department of Geography and School of Public Policy and Global Affairs University of British Columbia Vancouver Canada; ^3^ School of Zoology Tel‐Aviv University Tel Aviv Israel; ^4^ School of Forestry and Natural Environment Aristotle University of Thessaloniki Thessaloniki Greece; ^5^ Department of Ecology and Evolution University of Lausanne Lausanne Switzerland

**Keywords:** barn owl, conflict, conservation psychology, environmental peacebuilding, environmental psychology

## Abstract

Psychologists have long known that contact with and exposure to so‐called outgroup members can help overcome separation, including those resulting from entrenched conflicts. Getting to know each other facilitates understanding and fosters tolerance, whether we think of national, religious, cultural, socio‐economic or age divides. Thus, we here expand the concept of Environmental Peacebuilding to Environmental Bridgebuilding, because sharing knowledge and practices around nature conservation objectives offers opportunities to find common ground for individuals and groups. With Environmental Bridgebuilding, we are emphasising the connectivity effects of nature conservation planning and implementation. Due to our shared experiences, we take our work with the barn owl, *Tyto alba* , as an example to discuss Environmental Bridgebuilding. This choice does not limit Environmental Bridgebuilding to this species, on the contrary. Moreover, we discuss psychological factors in addition to between‐subgroup and intergroup challenges that might impact the success of socially engaging nature conservation initiatives, as well as the need for the combined expertise from various professions, such as biologists, psychologists, farmers and economists. Ecology and science more generally can be an inspiring source to bring together people that life has divided.

The time for seeking global solutions is running out. We can find suitable solutions only if we act together and in agreement. Pope Francis, 266th Catholic Pope

For scientists, it is a relief when prominent religious leaders join them in communicating that humans are the source of contemporary environmental problems and must be part of the solution. And, yes, understanding their own responsibility is for many a long way to go. In 2024, for instance, Gounaridis and Newell reported that about 15% of the US population denies climate change. The percentages of South American (Spektor et al. 2023) and European (Eichhorn and Grabbe 2025) countries are lower, but not far behind. Chapman and Peres (2026) highlighted the difficulty of combating denial and misconceptions around the damaging impact of deforestation and biodiversity loss. According to their analysis of the literature, effective conservation strategies cannot be implemented through rigorous research and publications in scientific journals only. The knowledge must reach the public in a comprehensive and ideally positive way (Milliet et al. 2024). To help steer human behaviour, the United Nations published its 17 Sustainable Developmental Goals (SDG) in 2015, but so far to limited avail (Ordonez‐Ponce 2023).

Accordingly, those who understand the importance of initiatives that counter the loss of biodiversity should continue in their efforts. The literature is full of examples that ring the alarm bell on endangered species on the verge of disappearing (see Lacher Jr et al. 2025 for a recent overview). These authors reported that more than 47,000 species are currently on the Red List of the International Union for Conservation of Nature, and of these over 10,000 are critically close to extinction. When insects, birds and vertebrates are monitored, dramatic population declines are observed across many regions of the world (Loiseau et al. 2020; Sánchez‐Bayo and Wyckhuys 2019). Certainly, less emblematic than elephants and lions, their disappearance directly harms our food systems and supply, economies, health and global stability (Díaz et al. 2019).

Stopping and reversing this loss requires a detailed understanding of its causes that are often associated with human activities, such as climate forcing emissions, the spread of diseases, pollution, habitat destruction, overexploitation, traffic and human conflicts (Daskin and Pringle 2018; Nikolaou and Katsanevakis 2023; Pereira et al. 2024; Singini and Baso 2025). In the current contribution, the literature on conflicts is particularly pertinent, because working together seems impossible when people and groups do not get along or fight against each other, possibly in violent ways. Then, people might find themselves stuck in an intractable situation of different values and perspectives, vested interests, path‐dependent materiality and the polarisation of discourses and positions (Lewicki et al. 2003; Lucas and Warman 2018).

And let us be real, having people care about biodiversity and nature conservation is already challenging enough. There are multifaceted factors that can facilitate pro‐environmental behaviour (Li et al. 2019; Piao and Managi 2024), but there are also many that are barriers (Dioba et al. 2024; Gifford 2011). Then, whatever factor has been determined in one context might not replicate in another, but depends on regional, societal, cultural, religious, anthropological or economic specificities (Dioba et al. 2024; Steg 2023). The same is true for the ways in which psychological functioning can ultimately lead people to change attitudes and behaviours towards nature conservation, and why (Clayton and Myers 2015; Fischer and Young 2007; Newell et al. 2014). This requires going beyond a rational choice approach relying on narrow valuations of biodiversity and ecosystem services to consider individuals' (ir)rational beliefs and religion (Minton et al. 2015), personal norms and values (Hopper and Nielsen 1991; Schoon and te Grotenhuis 2000; Tanner and Wölfing Kast 2003) as well as their personality (Soutter et al. 2020).

To provide some examples, changing individuals' illegal behaviour, such as poaching, might be difficult because these people already feel marginalised by society, having little motivation to comply with societal demands (Hübschle 2017). We better try to change the causes and feelings of marginalisation and injustice before the actual behaviour of poaching itself. In analogy, changes in spiritual beliefs may be more effective than prohibiting the spiritual routines of traditional medical practitioners and healers using the body parts of endangered animals, such as pangolins *Smutsia gigantea* (Boakye et al. 2014). Finally, not every individual needs to be targeted to positively influence their nature conservation behaviour. A meta‐analysis showed that the best personality predictors of pro‐environmental attitudes and behaviours are self‐reported openness and honesty humility, and to a lower extent agreeableness, conscientiousness and extraversion (Soutter et al. 2020). Initiatives to boost nature conservation behaviour might be lost on such individuals, because they likely need no further convincing.

Thus, it is complicated to have as many people as possible to engage in pro‐environmental behaviour. By default, this situation is worsened in times of conflict, when there is little place for common ground. Gladly, Environmental Peacebuilding (Dresse et al. 2019; Ide et al. 2021; Zelli and Krause 2025) has brought some hope to this otherwise bleak situation. Environmental Peacebuilding rests on the idea that environment‐related concerns and activities enable and reinforce constructive interactions between groups in conflict, in part because shared interests in the environment can be an incentive for cooperation and peace. This idea might sound contradictory, because nature and by inference territory, habitat and resources are frequently seen as sources of conflict rather than peace (Homer‐Dixon 1994; Le Billon 2013). Environmental Peacebuilding, however, works on the premise that peace and reconciliation can be achieved if the causes of conflict can be identified and constructive interactions between opponents can take place. Prominent examples are, for instance, the Nile Basin Initiative (https://nilebasin.org/) and the Peace Parks Foundation (https://www.peaceparks.org/).

There are, however, many more reasons than conflict to connect people through the shared benefits of their natural environment. We thus introduce the concept of *Environmental Bridgebuilding*. Environmental Peacebuilding and Environmental Bridgebuilding are closely related, but they are not identical. Environmental Peacebuilding mainly refers to environmental cooperation in contexts of actual, recent or threatened conflict, where shared ecological concerns may contribute to de‐escalation, dialogue, reconciliation or post‐conflict recovery. Environmental Bridgebuilding is broader. It refers to the capacity of nature conservation and environmental cooperation to create trust, communication, shared purpose and practical collaboration across existing social, professional, cultural, political or territorial divides, even where violent conflict is absent.

In this sense, Environmental Peacebuilding may be understood as one particular form of Environmental Bridgebuilding under conflict conditions. The leitmotif therein is the establishment of environmentally friendly initiatives to unite people, groups or even nations when facing common environmental challenges. In fact, Environmental Bridgebuilding could even be a way to prevent the rise of conflict by engaging in dialogue before it is too late.

Initiatives that combine the environment, development and the getting together of otherwise separated groups have emerged systematically since the end of the Cold War (Ali 2007; Hardt and Scheffran 2019). A recent and telling example is the worldwide spread of the Friday for Future movement, initiated by the young environmental activist Greta Thunberg. Her persona highlights how a passionate commitment to an environmental cause can move people's willingness to follow suite (Grabo and van Vugt 2016). Notably, there are other prominent initiatives, such as the Earth Hour (https://www.earthhour.org/), or the Plastic Free July (https://www.plasticfreejuly.org/) initiative. Worth mentioning, social initiative seems to benefit from symbols or a mascot with which its members can identify (Cayla 2013; Butler et al. 2020). In case of Friday for Future, we have Greta Thunberg herself. Then, Dame Jane Morris Goodall (1934–2025) pioneered research on wild chimpanzees. Still in our memory is the famous landmine walk by Lady Diana, the late Princess of Wales, in Angola. Her visibility brought a turning point in the fight against the lethal devices.

Such examples are inspiring to Environmental Bridgebuilding. We imagine many more initiatives that help create social identities, ideally represented through shared goals and symbols. The vision is that heterogeneous groups step beyond what formerly separated them (Hogg and Williams 2000). In the end, environmental challenges such as climate change and the loss of biodiversity are common threats, whoever the individual, whatever the attitudes or convictions of individuals within any given group. Important also, negative messaging has a stronger mobilising impact than positive ones (Young 2023). Theoretically speaking, this mix might help move people into cooperative collective action (Levin and Weber 2024). On the practical side, however, it is not easy to study and change human behaviour, neither on the individual nor on the group level (Palm et al. 2020; Butler et al. 1996; Oreg 2003). It is even worse when established beliefs and attitudes are implicated (Ecker et al. 2022; Kuhn et al. 2023; Lewandowsky 2021; Rafferty et al. 2013) as well as habits (Linder et al. 2022).

Overall, the literature paints a truly complicated situation on many levels. And yet, we want to give the concept of Environmental Bridgebuilding a fair chance. Thus, we here discuss different levels at which we suggest to favourably engage humans individually as well as in groups. In particular, we decided to distinguish between (i) individual, (ii) between‐subgroup and (iii) intergroup challenges. We elaborated our thought processes based on our work with the barn owl. By doing so, we do not wish to marginalise any other societal effort in nature conservation. All of them are needed. For instance, the Dinaric‐Balkan‐Pindos Large Carnivore Initiative protects the brown bear *Ursus arctos* , the wolf *Canis lupus* and the *Eurasian lynx* (https://dinaric‐carnivores.org/en/). The DMZ Forum (https://www.dmzforum.org) project aims to preserve vital crane habitats in and near the Korean Demilitarised Zone (DMZ) for the red‐crowned crane, *Grus japonensis* .

Returning to above three challenges, they are analytically distinct but closely connected. At the individual level, Environmental Bridgebuilding depends on beliefs, emotions, values, motivations and readiness to change. At the between‐subgroup level, it depends on communication, trust and coordination among actors with different expertise, interests and constraints, such as farmers, ecologists, educators, policy‐makers, or local communities. At the intergroup level, it concerns cooperation across broader social, cultural, territorial, or political divides, including settings marked by borders, historical tensions, or open conflict. Together, these levels help clarify how conservation can connect people not only symbolically, but also through practical, repeated and mutually beneficial forms of collaboration.

## 1 Barn Owls as a Test Case for Environmental Bridgebuilding

The barn owl provides a particularly suitable test case for Environmental Bridgebuilding because it sits at the intersection of ecology, agriculture, culture and cooperation. It is widely distributed, closely associated with open farmland and directly relevant to human livelihoods through its role as a natural predator of rodents. At the same time, it is a species surrounded by strong symbolic meanings, including fear and superstition in many cultural contexts. Its conservation therefore cannot rely on biology alone: it requires coordinated action among farmers, scientists, educators, local communities and sometimes actors across administrative or national borders. For these reasons, the barn owl offers a useful example of how conservation can generate both practical collaboration and social connection across different kinds of divides.

The barn owl is a naturally occurring nocturnal raptor. It can be observed worldwide, as long as it is not too cold and there are open fields to hunt (Roulin 2020). Indeed, this bird is regularly discussed as an efficient natural pest control agent, being ecologically and economically more sustainable than spreading rodenticides (Bontzorlos et al. 2024; Peleg et al. 2018; Kan et al. 2014). Among the major threats that barn owls encounter are superstitions, secondary poisoning, collisions with vehicles and habitat changes, including the loss of nest sites. For superstitions, an important proportion of people are scared by the bird, due to deeply engrained false beliefs, passed on from one to the next generation (Sebele et al. 2022). Many people are convinced that the owl's presence is a bad, even deadly, omen (see Buthelezi et al. 2026). Thus, for them, this bird needs to be avoided at all costs, and frequently, it means killing them. Regarding secondary poisoning, the industry developed efficient poisons to regulate rodent populations. Given that poisoned rodents do not die immediately, they continue to roam and are captured by predators who accumulate poison in their bodies and die. The poison is sufficiently harmful to affect predators such as barn owls (Roulin 2020).

Given this complex situation, it is important to work on people's superstitions, reduce rodenticide use and provide nesting opportunities. The dilemma, all three challenges need to be considered at once, because it will not suffice that one person fixes nest boxes, when one neighbour uses rodenticides, and the other one is scared of the bird and kills it at the next opportunity. The animal has not chosen or asked for our actions and beliefs but just breeds and flies where the habitat is apt for its survival, from one field to the next, irrespective of human ownership. Here, we face a perfect call for Environmental Bridgebuilding, because an individual alone cannot achieve the bird's protection and propagation in one's region to support rodent control. It is only possible when the effort is coordinated and shared across people and groups.

To further discuss this scenario, and for it to succeed, we discuss the three challenges on the individual (e.g., superstitions), between sub‐groups (e.g., vested interests of farmers, ecologists, builders) and intergroup (e.g., language, borders, war) level.

### 1.1 Individual Challenges

Psychological perspectives have become recognised as essential for effective conservation. Here, the focus lies on the individual, meaning on psychological factors and individual differences that may predict who is more or less likely to engage in conservation actions (see van Eeden et al. 2025 for a recent perspective). Conservation psychology addresses this by integrating psychological theories and methods into conservation practices (Saunders 2003; Clayton and Myers 2015). This young domain is outcome‐oriented, with the explicit goal of supporting biodiversity conservation and environmental sustainability.

Psychologists can contribute to understanding and shaping conservation behaviour and, hence, outcomes. Psychologists are trained in the development and application of various assessment and measurement tools such as questionnaires, interviews and behavioural tasks. They are also trained to evaluate the effectiveness of interventions aimed at behavioural change, and not only in the clinical domain (e.g., Michie et al. 2011). Identifying relevant psychological factors can help determine which individuals or groups are most likely to engage in conservation and which may benefit from targeted support (see also Clayton and Myers 2015; Stern 2000), something biologists are less aware of. Nature conservation is not restricted to nature lovers but concerns all humans.

Owls provide a useful example of how such an approach can be applied. Psychological studies can examine people's mental representations of owls and their protection, as well as their broader attitudes towards nature, animals and biodiversity. These representations can be studied in the general population or among specific stakeholder groups (e.g., farmers, see between subgroups challenges) (Theubet et al. 2025). Previous research suggests that person‐specific factors such as personality traits, belief in paranormal phenomena, gender, age, tradition and culture may shape environmental attitudes and behaviours (Gifford and Nilsson 2014). Examining these variables could help identify different psychological profiles of individuals holding predominantly positive versus negative representations of owls.

We started to investigate these questions several years ago. We launched an ongoing online survey, translated into 42 languages and distributed in nearly 150 countries. In it, we assess participants' socio‐demographic information (e.g., age, gender, having a pet), personality, attitudes and beliefs about the barn owl and proximity to nature (e.g., birdwatching). We recently reported on the Swiss data (Theubet et al. 2025) separating participants' answers into those that reflect positive versus negative attitudes towards the barn owl. Then, we compared the other variables between these groups. Results showed overall that Swiss participants have positive feelings towards the barn owl. Yet, younger individuals who do not consider themselves nature‐oriented, those who have never seen owls in the wild, and those who do not own pets are more likely to express negative feelings. Targeting these groups in outreach or educational interventions may, therefore, help promote the protection of these birds.

Admittedly, understanding these representations does not directly translate into behaviour or behavioural changes. Individuals may hold positive beliefs or feelings about owls without being motivated to protect them. However, the opposite situation may have more immediate consequences: if someone believes that the presence of an owl brings death or misfortune, they may attempt to kill it. In such cases, actions are guided by beliefs rather than facts. Changing beliefs, however, is difficult (Fang et al. 2017; Ecker et al. 2022), partly because people tend to seek information that confirms their existing views (confirmation bias) (Butera et al. 2018; Nickerson 1998). When confronted with contradictory information, individuals are more likely to assimilate rather than accommodate new knowledge (Block 1982; Piaget and Cook 1952). Moreover, such cognitive tendencies may apply more strongly to negative and threatening information than to positive information (Knobloch‐Westerwick et al. 2020). For these reasons, conservation initiatives must carefully consider both who is being targeted and how messages are communicated.

### 1.2 Between‐Subgroup Challenges

Social groups are collections of people whose members are interdependent, influencing one another through interactions and shared goals, and often common rules and norms. Individuals typically belong to several groups, such as family, music or sports communities, political affiliations, age groups and professions. These groups are diverse and have different interests, and thus, their willingness to protect nature varies (e.g., van Eeden et al. 2025). This also applies to our owl project, in which we are dealing with various not necessarily exclusive groups such as ornithologists, farmers, environmentalists and scientists. These groups care about the same environment but do not need to have the same vision of and a tradition for land management (e.g., Rac et al. 2024; Rust et al. 2022).

For obvious reasons, those who need soil and open space for their professional income assume that conservation limits human activity and economic growth (McKinnon et al. 2016; Milliet et al. 2024). This notion particularly affects farmers in a productivity‐driven sector reliant on yields, often prompting intensive agrochemical use (Le et al. 2023). A situation that is truly paradoxical, because society expects high yields alongside eco‐friendly methods, which usually implies lower output (Milliet et al. 2024). These incompatible demands create technical and psychological challenges, as farmers are often blamed for biodiversity loss (Dudley and Alexander 2017; Slingenberg et al. 2009). And it is a fact that traditional farming under efficiency pressure harms biodiversity (Geiger et al. 2010; McLaughlin and Mineau 1995), as seen in Central and Eastern Europe, where biodiversity dropped after European Community integration and changes in practices like pest control and pollination (Sutcliffe et al. 2015).

To reconcile competing interests, governments have promoted pro‐environmental farming, but farmland species such as birds continue to decline (Knaus and Antoniazza 2018; Rigal et al. 2023). Supporting farmers' conservation outcome requires economic incentives, understanding psychological motivators (Ezzine‐de‐Blas et al. 2019; Floress et al. 2017), and local dialogue with farmers self‐organising and participating in decisions to ensure practices are accepted (Oberč and Arroyo Schnell 2020; Milliet et al. 2024). But then, on the search for a feasible implementation, governments regularly change track, forcing farmers to adjust their practices again in short time windows (Huber et al. 2018; Kuehne et al. 2017). This is demotivating and counterproductive because farmers' understanding, support and cooperation are crucial for adopting new routines (Burrows and Kinney 2016; Hayat et al. 2020). Importantly, studies have also shown that informed farmers are more willing to change, highlighting the need for effective communication, trusted information sources and long‐term incentives like secure land tenure and market access (Gabel et al. 2018; Maas et al. 2021).

Moving away from pesticides and favouring biological pest control, such as the owl, is not done in a day, either. Moreover, using pesticides and owls concurrently is not possible, because owls will die when eating rodents that had ingested pesticides. Here, the different players need to understand each other and stay in dialogue to support farmers in their adaptation of owls in their vicinities. Large‐scale conservation of biodiversity is a prime challenge of the tragedy of the commons, as we all share our planet (Hardin 1968). Individuals and groups face a supposed incompatibility between economic growth and environmental protection (Rees 2003). The fact that we all have ‘free’ access to biodiversity ultimately reduces it because of over‐exploitation leading to its ultimate loss. Ostrom (2009), who received the Nobel Memorial Prize in Economic Sciences, puts it more positively, stating, ‘When natural resources are jointly used by their users, in time, rules are established for how these are to be cared for and used in a way that is both economically and ecologically sustainable’.

To best use our commons, we need knowledge, rules and collective actions (Levin and Weber 2024) as well as an understanding of how local behaviour might spread more widely (Tump et al. 2024). To empirically assess these, we can work with the Norm Activation Model (Schwartz 1977) as well as the Theory of Planned Behaviour (Ajzen 1991). For instance, if we want to know if people actively contribute to nature conservation, we need to know whether a person's intentions, norms, abilities and attitudes transform into actual behaviour. Both theories are regularly applied, because they proclaim that intentions, attitudes and norms predict behaviour. Encouraging, enhanced perceived collective efficacy as well as self‐efficacy increased pro‐environmental intentions among students (Jugert et al. 2016; Keshavarz and Karami 2016; Kropf et al. 2020). Also, environmental knowledge positively impacts pro‐environmental behaviours (Frick et al. 2004; Gabel et al. 2018; Meinhold and Malkus 2005). Most importantly, these models are also of relevance to the farming community. Three variables, Awareness of Consequences, Perceived Behavioural Control and Subjective Norms predicted pro‐environmental behavioural intention (Savari et al. 2023).

Regarding owls, we are dealing with professional and interest groups who rarely communicate, and if so, defend perspectives that are difficult to reconcile. How can farmers be motivated to engage in the conservation of biodiversity when being presented like adversaries, being denigrated, and rejected? Then, ecologists and farmers have different knowledge about agriculture and its impact on biodiversity. Only dialogue can result in combined knowledge, and by inference, common ground. Thus, in Environmental Bridgebuilding, supported by behavioural theories (Ajzen 1991; Schwartz 1977), we would have to work on behavioural intentions, shape personal attitudes and norms towards owl conservation, counter superstitions and provide knowledge and feelings of empowerment what can be done and how. For this to work, one would have to increase shared environmental knowledge and opportunities to communicate, fostering collaborations that are supported by all parties involved. Here, economic incentives could be helpful, such as more direct access to markets reducing predatory intermediaries. Also, one could increase the valuation of environmentally more benign food (e.g., organic) by consumers to financially incentivise farmers to shift to more environmentally friendly modes of production. And owls would fit here perfectly, because they can be used as biological pest control agents and are ecologically and economically more sustainable than spreading rodenticides (Kan et al. 2014).

Maintaining alive any nature conservation programmes in the long‐term requires broadly defined economic incentives (Authelet et al. 2021; Parkhurst and Shogren 2021). Thus, conservation initiatives should have lasting effects when yielding benefits to the local economies of group members involved in the implementation and sustainability of our approach (Emerton 2000). To maintain any programme, even if based on altruistic foundations, the benefit must be clear and the economic one is the most sustainable (Bottazzi et al. 2018; Jones et al. 2017; but see Reddy et al. 2017; Rajapaksa et al. 2019).

### 1.3 Intergroup Challenges

The discussion of the between‐subgroup level feeds into the intergroup challenges. We have made this separation, because of previous owl projects within the framework of Environmental Peacebuilding (Roulin et al. 2017). These projects can also be found under the label Owls for Peace (Roulin et al. 2017; https://www.chouette‐effraie.ch/en). Owls for Peace was initiated in the Middle East with the idea that owl conservation for agricultural benefit brings Israelis, Palestinians and Jordanians together. They all have to reduce their rodenticide use, create nesting opportunities for owls, monitor the boxes and share among them strategies and nesting box occupation. Together, they can now manage their shared natural reserve, because the bird does not know boundaries, hunting rodents in fields across borders. A healthy barn owl population in the region is of benefit to all, providing opportunities for positive dialogue among farmers as well as with the advising ecologists.

Owls for Peace, thus, solves an agroecological problem that requires a regional rather than a national solution. The project needs to engage neighbouring farmers to favour barn owls eating rodents instead of spreading tons of poison. This bottom‐up approach has been, and still is, supported by political leaders (top‐down), thereby helping its success in the Middle East (Roulin et al. 2017) and expanded into Cyprus involving the northern (Turkish) and southern parts (Greek) (Bontzorlos et al. 2024). Today, Owls for Peace is part of the International Ornithologists' Union (IOU) working group Birds as Peacemakers. Owls for Peace as an Environmental Peacebuilding initiative showed that nature conservation can promote communication and collaboration between societies that are otherwise separate. Sometimes, these societies might even be in conflict, armed or not. Indeed, there are about 227 peace parks around the world contributing to nature conservation while addressing political challenges (Ali 2007; Barquet et al. 2014; CDB 2018). Yet not all peace park initiatives have been successful (Barquet et al. 2014; Duffy 2001), including when countries cannot agree on the management of transboundary protected areas, or when one country tries to impose its agenda on others (van Amerom and Büscher 2005). There can also be tensions between different goals when pursuing ecological peace (peace with nature), social peace (peace between local communities) and international peace (peace between neighbouring countries) (Hsiao and Le Billon 2021).

The process of Environmental Peacebuilding requires interactions at various levels of society. Peace treaties imposed by third parties, such as regional and national politicians, or even the United Nations (top‐down approach) may not be followed if citizens are not ready for peace (Hemmer et al. 2006). If, however, citizens have already started to re‐engage in dialogue (bottom‐up approach), politicians may find ways to partake in peace negotiations. Both approaches require attention to person‐ and group‐specific variables, as well as to societal, historical, cultural and religious characteristics of communities in conflict (Ali 2007; Hardt and Scheffran 2019). Once contact has been established, reconciliation should be sustained through common goals that persist through time and necessitate investments that help (re)build trust and mutual well‐being, including improvement in local economies.

At the same time, Environmental Bridgebuilding should not be romanticised. In situations of acute violence or open war, conservation‐based cooperation may be interrupted, deprioritised, or simply rendered impossible. Nature conservation alone cannot overcome all the material, political, historical and emotional forces that sustain violent conflict. Its contribution may instead lie in helping to prevent escalation before violence intensifies, in maintaining limited channels of contact when conditions still allow, and in providing a basis for renewed cooperation when more favourable circumstances return. In this sense, Environmental Bridgebuilding is not a substitute for political solutions, but it may help preserve or rebuild relational conditions that make future dialogue more conceivable.

In Owls for Peace, we reunite people for regular meetings. The objective is to discuss how the use of barn owls as a biological pest control agent is implemented. For example, we learn how to analyse pellets to gather information about which animals owls consume (these pellets contain skulls and bones of the barn owls' prey, with which we can identify prey species). We also promote collaborative scientific research, for instance, through GPS data collection to improve environmental management. All this data is useful to raise awareness about the ecological roles of owls, not only among farming communities, but also among school children and politicians responsible for agricultural policies. The success is evident in Israel, where Jewish and Arab farmers work hand in hand to erect barn owl nesting boxes in their fields. This is also the case in Jordan and to a lesser extent in Palestine. To expand this initiative worldwide and to other birds, the IOU uses the terminology Birds as Peacemakers (https://www.internationalornithology.org/birds‐as‐peacemakers).

## 2 Conclusion

Taken together, the barn owl example suggests that Environmental Bridgebuilding can operate simultaneously across several levels. At the individual level, it requires attention to beliefs, emotions and willingness to change, especially where fear, superstition, or indifference shape human responses to a species. At the between‐subgroup level, it depends on communication, trust and coordination among actors with different forms of knowledge, interests and constraints. At the intergroup level, it can provide repeated opportunities for cooperation across broader territorial, cultural, or political divides. The case therefore illustrates how a conservation initiative may become not only ecologically relevant, but also socially connective.

Environmental Bridgebuilding suggests that nature conservation can do more than protect species and ecosystems; under appropriate conditions, it can also create trust, dialogue and cooperation among people who might otherwise remain socially, professionally, culturally, or politically separated. Its added value lies in drawing attention to conservation as a socially connective practice not only in conflict settings, but also in everyday contexts marked by distance, mistrust, or competing interests. We therefore propose Environmental Bridgebuilding as both a conceptual lens and a practical research agenda. Future work should examine more systematically under which conditions such initiatives succeed, for whom, through which mechanisms, and with what kinds of ecological and social outcomes. Further research could assess how durable these bridgebuilding effects are over time, how they interact with economic incentives, and how lessons from one conservation context may or may not transfer to another.

## Author Contributions

**Christine Mohr:** conceptualization (equal). **Philippe Le Billon:** conceptualization (equal). **Yossi Leshem:** conceptualization (equal). **Vasileios Bontzorlos:** conceptualization (equal). **Alexandre Roulin:** conceptualization (equal).

## Funding

The authors have nothing to report.

## Conflicts of Interest

The authors declare no conflicts of interest.

## Data Availability

The authors have nothing to report.
